# Supplementary material for: Transitions in metabolic syndrome and metabolic obesity status over time and risk of urologic cancer: A prospective cohort study
Source: PLoS One. 2024 Oct 21;19(10):e0311492. doi: 10.1371/journal.pone.0311492 (PMC11493304; doi:10.1371/journal.pone.0311492)
Supplement: S1 Table — (DOCX) [file pone.0311492.s001.docx]

S1 Table. Characteristics of participants by transitions in MetS status, 2006-2007 to 2008-2009.

| Characteristics | Non-MetS(2006-2007) | | MetS(2006-2007) | |
| --- | --- | --- | --- | --- |
|  | Non-MetS(2008-2009)  (n=32826) | MetS(2008-2009)  (n=9905) | Non-MetS(2008-2009)  (n=7025) | MetS(2008-2009)  (n=12963) |
| Age(years,mean±SD) | 50.52±12.54 | 53.73±11.77 | 54.23±11.60 | 55.74±10.77 |
| Gender, n(%) |  |  |  |  |
| Female | 6940(21.14) | 1734(17.51) | 1139(16.21) | 2476(19.10) |
| Male | 25886(78.86) | 8171(82.49) | 5886(83.79) | 10487(80.90) |
| Smoking status, n(%) |  |  |  |  |
| Never | 19397(59.09) | 5716(57.71) | 3991(56.81) | 7501(57.86) |
| Former | 1510(4.60) | 573(5.78) | 492(7.00) | 898(6.93) |
| Current | 11919(36.31) | 3616(36.51) | 2542(36.19) | 4564(35.21) |
| Alcohol consumption, n(%) |  |  |  |  |
| Never | 19039(58.00) | 5539(55.92) | 4003(56.98) | 7418(57.22) |
| Former | 1029(3.13) | 361(3.64) | 326(4.64) | 582(4.49) |
| Current | 12758(38.87) | 4005(40.43) | 2696(38.38) | 4963(38.29) |
| Occupation, n(%) |  |  |  |  |
| White collar | 2081(6.34) | 641(6.47) | 420(5.98) | 958(7.39) |
| Blue collar | 30745(93.66) | 9264(93.53) | 6605(94.02) | 12005(92.61) |
| Education level, n(%) |  |  |  |  |
| Illiteracy and primary | 2931(8.93) | 1003(10.13) | 914(13.01) | 1611(12.43) |
| Middle school | 28009(85.33) | 8442(85.23) | 5872(83.59) | 10829(83.54) |
| College and above | 1886(5.75) | 460(4.64) | 239(3.40) | 523(4.03) |
| Income(yuan per psrson per month) , n(%) | |  |  |  |
| <600 | 10197(31.06) | 2881(29.09) | 2341(33.32) | 3985(30.74) |
| ≥600-<1000 | 20454(62.31) | 6355(64.16) | 4224(60.13) | 8051(62.11) |
| ≥1000 | 2175(6.63) | 669(6.75) | 460(6.55) | 927(7.15) |
| Marital status, n(%) |  |  |  |  |
| Single | 1552(4.73) | 370(3.74) | 253(3.60) | 423(3.26) |
| Married/cohabiting | 31274(95.27) | 9535(96.26) | 6772(96.40) | 12540(96.74) |
| Salt intake, n(%) |  |  |  |  |
| Light | 3215(9.79) | 922(9.31) | 752(10.70) | 1233(9.51) |
| General | 26271(80.03) | 7865(79.40) | 5454(77.64) | 10015(77.26) |
| Heavy | 3340(10.17) | 1118(11.29) | 819(11.66) | 1715(13.23) |
| Sitting time(h/day), n(%) |  |  |  |  |
| <4 | 24620(75.00) | 7485(75.57) | 5325(75.80) | 9529(73.51) |
| ≥4-<8 | 7248(22.08) | 2177(21.98) | 1504(21.41) | 3042(23.47) |
| ≥8 | 958(2.92) | 243(2.45) | 196(2.79) | 392(3.02) |

Abbreviations: MetS, metabolic syndrome.
