# Supplementary material for: Transitions in metabolic syndrome and metabolic obesity status over time and risk of urologic cancer: A prospective cohort study
Source: PLoS One. 2024 Oct 21;19(10):e0311492. doi: 10.1371/journal.pone.0311492 (PMC11493304; doi:10.1371/journal.pone.0311492)
Supplement: S2 Table — (DOCX) [file pone.0311492.s002.docx]

S2 Table. Baseline characteristics of participants by MO status, 2006-2007.

| Characteristics | MHN  (n=59385) | MHO  (n=7176) | MUN  (n=20290) | MUO  (n=11046) |
| --- | --- | --- | --- | --- |
| Age(years,mean±SD) | 50.55±12.90 | 49.66±112.51 | 55.39±11.18 | 52.91±11.66 |
| Gender, n(%) |  |  |  |  |
| Female | 12443(20.95) | 1526(21.27) | 3742(18.44) | 1901(17.21) |
| Male | 46942(79.05) | 5650(78.73) | 16548(81.56) | 9145(82.79) |
| Smoking status, n(%) |  |  |  |  |
| Never | 35534(59.84) | 4631(64.53) | 11826(58.28) | 6336(57.36) |
| Former | 3059(5.15) | 404(5.63) | 1511(7.45) | 893(8.08) |
| Current | 20792(35.01) | 2141(29.84) | 6953(34.27) | 3817(34.56) |
| Alcohol consumption, n(%) |  |  |  |  |
| Never | 34893(58.76) | 4439(61.86) | 11810(58.21) | 6300(57.03) |
| Former | 2120(3.57) | 242(3.37) | 1074(5.29) | 567(5.13) |
| Current | 22372(37.67) | 2495(34.77) | 7406(36.50) | 4179(37.83) |
| Occupation, n(%) |  |  |  |  |
| White collar | 4811(8.10) | 458(6.38) | 1729(8.52) | 886(8.02) |
| Blue collar | 54574(91.90) | 6718(93.62) | 18561(91.48) | 10160(91.98) |
| Education level, n(%) |  |  |  |  |
| Illiteracy and primary | 6035(10.16) | 672(9.36) | 2777(13.69) | 1480(13.40) |
| Middle school | 48541(81.74) | 6019(83.88) | 16466(81.15) | 8875(80.35) |
| College and above | 4809(8.10) | 485(6.76) | 1047(5.16) | 691(6.26) |
| Income(yuan per psrson per month) , n(%) | |  |  |  |
| <600 | 16671(28.07) | 1904(26.53) | 5858(28.87) | 3464(31.36) |
| ≥600-<1000 | 38119(64.19) | 4762(66.36) | 12830(63.23) | 6699(60.65) |
| ≥1000 | 4595(7.74) | 510(7.11) | 1602(7.90) | 883(7.99) |
| Marital status, n(%) |  |  |  |  |
| Single | 2898(4.88) | 299(4.17) | 748(3.69) | 475(4.30) |
| Married/cohabiting | 56487(95.12) | 6877(95.83) | 19542(96.31) | 10571(95.70) |
| Salt intake, n(%) |  |  |  |  |
| Light | 5678(9.56) | 562(7.83) | 1954(9.63) | 1007(9.12) |
| General | 47777(80.45) | 5858(81.63) | 15997(78.84) | 8490(76.86) |
| Heavy | 5930(9.99) | 756(10.54) | 2339(11.53) | 1549(14.02) |
| Sitting time(h/day), n(%) |  |  |  |  |
| <4 | 44348(74.68) | 5373(74.87) | 15070(74.27) | 7923(71.73) |
| ≥4-<8 | 13189(22.21) | 1557(21.70) | 4618(22.76) | 2706(24.50) |
| ≥8 | 1848(3.11) | 246(3.43) | 602(2.97) | 417(3.78) |

Abbreviations: MO, metabolic obesity; MHN, metabolically healthy normal weight; MHO, metabolically healthy obesity; MUN, metabolically unhealthy normal weight; MUO, metabolically unhealthy obesity.
