# Supplementary material for: Transitions in metabolic syndrome and metabolic obesity status over time and risk of urologic cancer: A prospective cohort study
Source: PLoS One. 2024 Oct 21;19(10):e0311492. doi: 10.1371/journal.pone.0311492 (PMC11493304; doi:10.1371/journal.pone.0311492)
Supplement: S3 Table — (DOCX) [file pone.0311492.s003.docx]

S3 Table. Characteristics of participants by transitions in MO status, 2006-2007 to 2008-2009.

| Characteristics | MHN(2006-2007) | MHO(2006-2007) | | MUN(2006-2007) | MUO(2006-2007) | | |
| --- | --- | --- | --- | --- | --- | --- | --- |
|  | MHN(2008-2009)  (n=29218) | MHO(2008-2009)  (n=1437) | MUO(2008-2009)  (n=1423) | MUO(2008-2009)  (n=958) | MHO(2008-2009)  (n=1005) | MUN(2008-2009)  (n=1355) | MUO(2008-2009)  (n=3992) |
| Age(years,mean±SD) | 50.64±12.54 | 49.01±12.40 | 51.41±12.51 | 57.10±11.08 | 51.69±12.62 | 55.18±10.67 | 53.84±11.30 |
| Gender, n(%) |  |  |  |  |  |  |  |
| Female | 6071  (20.78) | 381  (26.51) | 276  (19.40) | 213  (22.23) | 174  (17.31) | 212  (15.65) | 708  (17.74) |
| Male | 23147  (79.22) | 1056  (73.49) | 1147  (80.60) | 745  (77.77) | 831  (82.69) | 1143  (84.35) | 3284  (82.26) |
| Smoking status, n(%) | |  |  |  |  |  |  |
| Never | 17051  (58.36) | 934  (65.00) | 865  (60.79) | 556  (58.04) | 575  (57.21) | 787  (58.08) | 2262  (56.66) |
| Former | 1344  (4.60) | 64  (4.45) | 102  (7.17) | 88  (9.19) | 80  (7.96) | 90  (6.64) | 293  (7.34) |
| Current | 10823  (37.04) | 439  (30.55) | 456  (32.04) | 314  (32.78) | 350  (34.83) | 478  (35.28) | 1437  (36.00) |
| Alcohol consumption, n(%) | |  |  |  |  |  |  |
| Never | 16768  (57.39) | 898  (62.49) | 823  (57.84) | 549  (57.31) | 577  (57.41) | 795  (58.67) | 2225  (55.74) |
| Former | 915  (3.13) | 46  (3.20) | 54  (3.79) | 43  (4.49) | 59  (5.87) | 54  (3.99) | 171  (4.28) |
| Current | 11535  (39.48) | 493  (34.31) | 546  (38.37) | 366  (38.20) | 369  (36.72) | 506  (37.34) | 1596  (39.98) |
| Occupation, n(%) |  |  |  |  |  |  |  |
| White collar | 1890  (6.47) | 78  (5.43) | 86  (6.04) | 87  (9.08) | 62  (6.17) | 80  (5.90) | 282  (7.06) |
| Blue collar | 27328  (93.53) | 1359  (94.57) | 1337  (93.96) | 871  (90.92) | 943  (93.83) | 1275  (94.10) | 3710  (92.94) |
| Education level, n(%) | |  |  |  |  |  |  |
| Illiteracy and primary | 2613  (8.94) | 124  (8.63) | 121  (8.50) | 135  (14.09) | 132  (13.13) | 143  (10.55) | 522  (13.08) |
| Middle school | 24900  (85.22) | 1235  (85.94) | 1234  (86.72) | 784  (81.84) | 826  (82.19) | 1160  (85.61) | 3280  (82.16) |
| College and above | 1705  (5.84) | 78  (5.43) | 68  (4.78) | 39  (4.07) | 47  (4.68) | 52  (3.84) | 190  (4.76) |
| Income(yuan per psrson per month) , n(%) | | |  |  |  |  |  |
| <600 | 9154  (31.33) | 419  (29.16) | 424  (29.80) | 281  (29.33) | 379  (37.71) | 404  (29.82) | 1370  (34.32) |
| ≥600-<1000 | 18106  (61.97) | 919  (63.95) | 902  (63.39) | 601  (62.73) | 569  (56.62) | 835  (61.62) | 2353  (58.94) |
| ≥1000 | 1958  (6.70) | 99  (6.89) | 97  (6.82) | 76  (7.93) | 57  (5.67) | 116  (8.56) | 269  (6.74) |
| Marital status, n(%) | |  |  |  |  |  |  |
| Single | 1401  (4.79) | 64  (4.45) | 63  (4.43) | 33  (3.44) | 50  (4.98) | 42  (3.10) | 161  (4.03) |
| Married/ cohabiting | 27817  (95.21) | 1373  (95.55) | 1360  (95.57) | 925  (96.56) | 955  (95.02) | 1313  (96.90) | 3831  (95.97) |
| Salt intake, n(%) |  |  |  |  |  |  |  |
| Light | 2912  (9.97) | 124  (8.63) | 116  (8.15) | 100  (10.44) | 101  (10.05) | 134  (9.89) | 366  (9.17) |
| General | 23332  (79.85) | 1160  (80.72) | 1124  (78.99) | 737  (76.93) | 765  (76.12) | 1049  (77.42) | 3020  (75.65) |
| Heavy | 2974  (10.18) | 153  (10.65) | 183  (12.86) | 121  (12.63) | 139  (13.83) | 172  (12.69) | 606  (15.18) |
| Sitting time(h/day), n(%) | |  |  |  |  |  |  |
| <4 | 21909  (74.98) | 1046  (72.79) | 1059  (74.42) | 712  (74.32) | 708  (70.45) | 1028  (75.87) | 2828  (70.84) |
| ≥4-<8 | 6477  (22.17) | 338  (23.52) | 323  (22.70) | 217  (22.65) | 253  (25.17) | 291  (21.48) | 1022  (25.60) |
| ≥8 | 832  (2.85) | 53  (3.69) | 41  (2.88) | 29  (3.03) | 44  (4.38) | 36  (2.66) | 142  (3.56) |

Abbreviations: MO, metabolic obesity; MHN, metabolically healthy normal weight; MHO, metabolically healthy obesity; MUN, metabolically unhealthy normal weight; MUO, metabolically unhealthy obesity.
