# Supplementary material for: Transitions in metabolic syndrome and metabolic obesity status over time and risk of urologic cancer: A prospective cohort study
Source: PLoS One. 2024 Oct 21;19(10):e0311492. doi: 10.1371/journal.pone.0311492 (PMC11493304; doi:10.1371/journal.pone.0311492)
Supplement: S4 Table — (DOCX) [file pone.0311492.s004.docx]

S4 Table. Subgroup analyses of the association between MetS status at baseline (2006-2007) and risk of UC.

| Variable | | Total  cases | Person  years | Incident  cases | HR(95%CI) | *P* for  interaction |
| --- | --- | --- | --- | --- | --- | --- |
| Age(years) |  |  |  |  |  | 0.010 |
| <55 | Non-MetS | 43663 | 598368.32 | 94 | Ref |  |
|  | MetS | 16762 | 227750.85 | 74 | 1.66(1.22-2.26) |  |
| ≥55 | Non-MetS | 22898 | 288016.84 | 238 | ref |  |
|  | MetS | 14574 | 178547.07 | 148 | 1.09(0.89-1.34) |  |
| Gender |  |  |  |  |  | 0.119 |
| Female | Non-MetS | 13969 | 192054.40 | 24 | Ref |  |
|  | MetS | 5643 | 75808.70 | 11 | 0.78(0.37-1.63) |  |
| Male | Non-MetS | 52592 | 694330.76 | 308 | ref |  |
|  | MetS | 25693 | 330489.23 | 211 | 1.31(1.10-1.56) |  |
| Smoking status |  |  |  |  |  | 0.573 |
| Never | Non-MetS | 40165 | 535986.52 | 172 | Ref |  |
|  | MetS | 18162 | 235470.67 | 110 | 1.20(0.94-1.53) |  |
| Former and current | Non-MetS | 26396 | 350398.64 | 160 | Ref |  |
|  | MetS | 13174 | 170827.26 | 112 | 1.32(1.04-1.69) |  |

Abbreviations: MetS, metabolic syndrome; UC, urologic cancer; HR, hazard ratio; CI, conﬁdence interval; Ref, reference.

Model was adjusted for age, gender, smoking status, alcohol consumption, occupation, education level, income, marital status, salt intake and sitting time.
