# Supplementary material for: Transitions in metabolic syndrome and metabolic obesity status over time and risk of urologic cancer: A prospective cohort study
Source: PLoS One. 2024 Oct 21;19(10):e0311492. doi: 10.1371/journal.pone.0311492 (PMC11493304; doi:10.1371/journal.pone.0311492)
Supplement: S5 Table — (DOCX) [file pone.0311492.s005.docx]

S5 Table. Subgroup analyses of the association between MO status at baseline (2006-2007) and risk of UC.

| Variable | | Total  cases | Person  years | Incident  cases | HR  (95%CI) | *P* for  interaction |
| --- | --- | --- | --- | --- | --- | --- |
| Age(years) |  |  |  |  |  | 0.013 |
| <55 | MHN | 38836 | 532186.56 | 85 | Ref |  |
|  | MHO | 4827 | 66181.76 | 9 | 0.90(0.45-1.80) |  |
|  | MUN | 10453 | 141951.18 | 42 | 1.43(0.99-2.08) |  |
|  | MUO | 6309 | 85799.67 | 32 | 2.04(1.36-3.06) |  |
| ≥55 | MHN | 20549 | 258095.64 | 218 | Ref |  |
|  | MHO | 2349 | 29921.19 | 20 | 0.88(0.56-1.39) |  |
|  | MUN | 9837 | 120014.73 | 94 | 1.00(0.78-1.27) |  |
|  | MUO | 4737 | 58532.35 | 54 | 1.24(0.92-1.67) |  |
| Gender |  |  |  |  |  | 0.076 |
| Female | MHN | 12443 | 171048.78 | 22 | Ref |  |
|  | MHO | 1526 | 21005.62 | 2 | 0.64(0.15-2.74) |  |
|  | MUN | 3742 | 50311.84 | 8 | 0.78(0.34-1.82) |  |
|  | MUO | 1901 | 25496.85 | 3 | 0.64(0.19-2.18) |  |
| Male | MHN | 46942 | 619233.43 | 281 | Ref |  |
|  | MHO | 5650 | 75097.33 | 27 | 0.91(0.61-1.35) |  |
|  | MUN | 16548 | 211654.06 | 128 | 1.16(0.94-1.44) |  |
|  | MUO | 9145 | 118835.17 | 83 | 1.57(1.23-2.01) |  |
| Smoking status | |  |  |  |  | 0.525 |
| Never | MHN | 35534 | 473838.71 | 156 | Ref |  |
|  | MHO | 4631 | 62147.81 | 16 | 0.84(0.50-1.41) |  |
|  | MUN | 11826 | 152728.81 | 68 | 1.07(0.81-1.43) |  |
|  | MUO | 6336 | 82741.86 | 42 | 1.40(0.99-1.97) |  |
| Former and current | MHN | 23851 | 316443.49 | 147 | Ref |  |
|  | MHO | 2545 | 33955.15 | 13 | 0.94(0.53-1.65) |  |
|  | MUN | 8464 | 109237.10 | 68 | 1.18(0.89-1.58) |  |
|  | MUO | 4710 | 61590.16 | 44 | 1.59(1.14-2.23) |  |

Abbreviations: MO, metabolic obesity; UC, urologic cancer; MHN, metabolically healthy normal weight; MHO, metabolically healthy obesity; MUN, metabolically unhealthy normal weight; MUO, metabolically unhealthy obesity; HR, hazard ratio; CI, conﬁdence interval; Ref, reference.

Model was adjusted for age, gender, smoking status, alcohol consumption, occupation, education level, income, marital status, salt intake and sitting time.
