# Supplementary material for: Transitions in metabolic syndrome and metabolic obesity status over time and risk of urologic cancer: A prospective cohort study
Source: PLoS One. 2024 Oct 21;19(10):e0311492. doi: 10.1371/journal.pone.0311492 (PMC11493304; doi:10.1371/journal.pone.0311492)
Supplement: S6 Table — (DOCX) [file pone.0311492.s006.docx]

S6 Table. Subgroup analyses of the association between transitions in MetS status (2006-2007 to 2008-2009) and risk of UC.

| Variable |  |  | Total  cases | Person  years | Incident  cases | HR(95%CI) | *P* for  interaction |
| --- | --- | --- | --- | --- | --- | --- | --- |
|  | MetS status  at baseline  (2006-2007) | MetS status  at follow-up  (2008-2009) |  |  |  |  |  |
| Age(years) |  |  |  |  |  |  | 0.022 |
| <55 | Non-MetS | Non-MetS | 21562 | 256995.23 | 36 | Ref |  |
|  | Non-MetS | MetS | 5531 | 65377.68 | 10 | 0.94(0.47-1.90) |  |
|  | MetS | Non-MetS | 3805 | 45164.35 | 12 | 1.59(0.82-3.05) |  |
|  | MetS | MetS | 6201 | 72821.58 | 26 | 1.99(1.20-3.30) |  |
| ≥55 | Non-MetS | Non-MetS | 11264 | 121957.45 | 103 | Ref |  |
|  | Non-MetS | MetS | 4373 | 47333.44 | 31 | 0.81(0.54-1.21) |  |
|  | MetS | Non-MetS | 3220 | 33812.90 | 32 | 1.18(0.79-1.75) |  |
|  | MetS | MetS | 6762 | 71459.08 | 66 | 1.25(0.92-1.70) |  |
| Gender |  |  |  |  |  |  | 0.061 |
| Female | Non-MetS | Non-MetS | 6940 | 81831.49 | 13 | Ref |  |
|  | Non-MetS | MetS | 1734 | 20195.36 | 1 | 0.21(0.03-1.65) |  |
|  | MetS | Non-MetS | 1139 | 13212.94 | 1 | 0.35(0.05-2.72) |  |
|  | MetS | MetS | 2476 | 28176.48 | 5 | 0.66(0.22-1.97) |  |
| Male | Non-MetS | Non-MetS | 25886 | 297121.20 | 126 | Ref |  |
|  | Non-MetS | MetS | 8171 | 92515.76 | 40 | 0.93(0.65-1.32) |  |
|  | MetS | Non-MetS | 5886 | 65764.31 | 43 | 1.40(0.99-1.97) |  |
|  | MetS | MetS | 10487 | 116104.18 | 87 | 1.55(1.18-2.03) |  |
| Smoking status | |  |  |  |  |  | 0.258 |
| Never | Non-MetS | Non-MetS | 19397 | 224126.96 | 76 | Ref |  |
|  | Non-MetS | MetS | 5716 | 64979.36 | 21 | 0.75(0.46-1.21) |  |
|  | MetS | Non-MetS | 3991 | 44819.75 | 15 | 0.81(0.47-1.41) |  |
|  | MetS | MetS | 7501 | 83319.69 | 48 | 1.33(0.92-1.91) |  |
| Former and current | Non-MetS | Non-MetS | 13429 | 154825.72 | 63 | Ref |  |
|  | Non-MetS | MetS | 4189 | 47731.75 | 20 | 0.99(0.59-1.62) |  |
|  | MetS | Non-MetS | 3034 | 34157.50 | 29 | 1.85(1.19-2.88) |  |
|  | MetS | MetS | 5462 | 60960.97 | 44 | 1.59(1.08-2.34) |  |

Abbreviations: MetS, metabolic syndrome; UC, urologic cancer; HR, hazard ratio; CI, conﬁdence interval; Ref, reference.

Model was adjusted for age, gender, smoking status, alcohol consumption, occupation, education level, income, marital status, salt intake and sitting time.
