# Supplementary material for: Transitions in metabolic syndrome and metabolic obesity status over time and risk of urologic cancer: A prospective cohort study
Source: PLoS One. 2024 Oct 21;19(10):e0311492. doi: 10.1371/journal.pone.0311492 (PMC11493304; doi:10.1371/journal.pone.0311492)
Supplement: S7 Table — (DOCX) [file pone.0311492.s007.docx]

S7 Table. Subgroup analyses of the association between transitions in MO status (2006-2007 to 2008-2009) and risk of UC.

| Variable |  |  | Total  cases | Person  years | Incident  cases | HR(95%CI) | *P* for  interaction |
| --- | --- | --- | --- | --- | --- | --- | --- |
|  | MO status  at baseline  (2006-2007) | MO status  at follow-up  (2008-2009) |  |  |  |  |  |
| Age(years) |  |  |  |  |  |  | 0.132 |
| <55 | MHN | MHN | 19149 | 228340.17 | 31 | Ref |  |
|  | MHO | MHO | 967 | 11493.46 | 1 | 0.76(0.10-5.54) |  |
|  | MHO | MUO | 866 | 10241.14 | 3 | 2.23(0.68-7.31) |  |
|  | MUN | MUO | 378 | 4389.33 | 1 | 1.35(0.18-9.89) |  |
|  | MUO | MHO | 596 | 7104.99 | 3 | 3.12(0.95-10.21) |  |
|  | MUO | MUN | 678 | 8028.43 | 2 | 1.35(0.32-5.65) |  |
|  | MUO | MUO | 2087 | 24570.08 | 10 | 2.55(1.25-5.22) |  |
| ≥55 | MHN | MHN | 10069 | 108907.78 | 90 | Ref |  |
|  | MHO | MHO | 470 | 5230.06 | 3 | 0.86(0.27-2.73) |  |
|  | MHO | MUO | 557 | 6204.65 | 5 | 1.09(0.44-2.69) |  |
|  | MUN | MUO | 580 | 6113.77 | 6 | 1.33(0.58-3.04) |  |
|  | MUO | MHO | 409 | 4338.29 | 8 | 2.47(1.19-5.10) |  |
|  | MUO | MUN | 677 | 7125.75 | 8 | 1.52(0.74-3.13) |  |
|  | MUO | MUO | 1905 | 20327.45 | 18 | 1.24(0.75-2.07) |  |
| Gender |  |  |  |  |  |  | 0.227 |
| Female | MHN | MHN | 6071 | 71655.76 | 9 | Ref |  |
|  | MHO | MHO | 381 | 4478.05 | 1 | 1.51(0.19-12.20) |  |
|  | MHO | MUO | 276 | 3223.78 | 0 | - |  |
|  | MUN | MUO | 213 | 2410.10 | 0 | - |  |
|  | MUO | MHO | 174 | 2019.75 | 0 | - |  |
|  | MUO | MUN | 212 | 2354.82 | 1 | 1.86(0.23-15.28) |  |
|  | MUO | MUO | 708 | 8135.63 | 1 | 0.61(0.07-4.97) |  |
| Male | MHN | MHN | 23147 | 265592.20 | 112 | Ref |  |
|  | MHO | MHO | 1056 | 12245.47 | 3 | 0.70(0.22-2.22) |  |
|  | MHO | MUO | 1147 | 13222.02 | 8 | 1.50(0.73-3.08) |  |
|  | MUN | MUO | 745 | 8093.00 | 7 | 1.58(0.74-3.40) |  |
|  | MUO | MHO | 831 | 9423.53 | 11 | 2.89(1.55-5.37) |  |
|  | MUO | MUN | 1143 | 12799.37 | 9 | 1.54(0.78-3.04) |  |
|  | MUO | MUO | 3284 | 36761.90 | 27 | 1.72(1.12-2.62) |  |
| Smoking status | |  |  |  |  |  | 0.221 |
| Never | MHN | MHN | 17051 | 196945.11 | 68 | Ref |  |
|  | MHO | MHO | 934 | 10883.29 | 1 | 0.32(0.04-2.28) |  |
|  | MHO | MUO | 865 | 10011.66 | 4 | 1.02(0.37-2.81) |  |
|  | MUN | MUO | 556 | 6098.15 | 4 | 1.48(0.54-4.07) |  |
|  | MUO | MHO | 575 | 6571.06 | 2 | 0.88(0.22-3.58) |  |
|  | MUO | MUN | 787 | 8683.17 | 7 | 1.82(0.84-3.96) |  |
|  | MUO | MUO | 2262 | 25348.10 | 13 | 1.30(0.72-2.36) |  |
| Former and current | MHN | MHN | 12167 | 140302.85 | 53 | Ref |  |
|  | MHO | MHO | 503 | 5840.22 | 3 | 1.67(0.52-5.34) |  |
|  | MHO | MUO | 558 | 6434.13 | 4 | 1.74(0.63-4.82) |  |
|  | MUN | MUO | 402 | 4404.96 | 3 | 1.32(0.41-4.25) |  |
|  | MUO | MHO | 430 | 4872.21 | 9 | 4.80(2.36-9.75) |  |
|  | MUO | MUN | 568 | 6471.01 | 3 | 1.18(0.37-3.77) |  |
|  | MUO | MUO | 1730 | 19549.43 | 15 | 2.03(1.14-3.60) |  |

Abbreviations: MO, metabolic obesity; UC, urologic cancer; MHN, metabolically healthy normal weight; MHO, metabolically healthy obesity; MUN, metabolically unhealthy normal weight; MUO, metabolically unhealthy obesity; HR, hazard ratio; CI, conﬁdence interval; Ref, reference.

Model was adjusted for age, gender, smoking status, alcohol consumption, occupation, education level, income, marital status, salt intake and sitting time.
