# Supplementary material for: Transitions in metabolic syndrome and metabolic obesity status over time and risk of urologic cancer: A prospective cohort study
Source: PLoS One. 2024 Oct 21;19(10):e0311492. doi: 10.1371/journal.pone.0311492 (PMC11493304; doi:10.1371/journal.pone.0311492)
Supplement: S8 Table — (DOCX) [file pone.0311492.s008.docx]

S8 Table. Sensitivity analyses of the association between MetS and MO status at baseline (2006-2007) and risk of UC.

| Group | | Total  cases | Person  years | Incident  cases | HR(95%CI) |
| --- | --- | --- | --- | --- | --- |
| Excluding participants with history of myocardial infarction and stroke. | | | | | |
| MetS status | Non-MetS | 64956 | 867856.24 | 313 | Ref |
|  | MetS | 29742 | 388364.41 | 207 | 1.28(1.08-1.53) |
| MO status | MHN | 57984 | 774240.11 | 285 | Ref |
|  | MHO | 6972 | 93616.13 | 28 | 0.92(0.62-1.36) |
|  | MUN | 19269 | 250593.16 | 128 | 1.16(0.94-1.43) |
|  | MUO | 10473 | 137771.25 | 79 | 1.50(1.17-1.93) |
| Excluding participants who developed urologic cancer within the first 2 years of follow-up. | | | | | |
| MetS status | Non-MetS | 66522 | 886348.25 | 293 | Ref |
|  | MetS | 31311 | 406273.43 | 197 | 1.28(1.06-1.53) |
| MO status | MHN | 59348 | 790246.33 | 266 | Ref |
|  | MHO | 7174 | 96101.93 | 27 | 0.94(0.63-1.39) |
|  | MUN | 20273 | 261951.19 | 119 | 1.13(0.91-1.41) |
|  | MUO | 11038 | 144322.24 | 78 | 1.55(1.20-1.99) |

Abbreviations: MetS, metabolic syndrome; MO, metabolic obesity; UC, urologic cancer; MHN, metabolically healthy normal weight; MHO, metabolically healthy obesity; MUN, metabolically unhealthy normal weight; MUO, metabolically unhealthy obesity; HR, hazard ratio; CI, conﬁdence interval; Ref, reference.

Model was adjusted for age, gender, smoking status, alcohol consumption, occupation, education level, income, marital status, salt intake and sitting time.
