# Supplementary material for: Transitions in metabolic syndrome and metabolic obesity status over time and risk of urologic cancer: A prospective cohort study
Source: PLoS One. 2024 Oct 21;19(10):e0311492. doi: 10.1371/journal.pone.0311492 (PMC11493304; doi:10.1371/journal.pone.0311492)
Supplement: S9 Table — (DOCX) [file pone.0311492.s009.docx]

S9 Table. Sensitivity analyses of the association between transitions in MetS and MO status (2006-2007 to 2008-2009) and risk of UC.

| Group | | |  | Total  cases | Person  years | Incident  cases | HR(95%CI) |
| --- | --- | --- | --- | --- | --- | --- | --- |
|  | | MO status  at baseline  (2006-2007) | MO status  at follow-up  (2008-2009) |  |  |  |  |
| Excluding participants with a history of myocardial infarction and stroke | | | | | | | |
| MetS status | Non-MetS | | Non-MetS | 32190 | 372353.35 | 133 | Ref |
|  | Non-MetS | | MetS | 9511 | 108610.76 | 40 | 0.89(0.63-1.27) |
|  | MetS | | Non-MetS | 6771 | 76469.53 | 43 | 1.34(0.95-1.90) |
|  | MetS | | MetS | 12188 | 136531.67 | 83 | 1.44(1.09-1.89) |
| MO status | MHN | | MHN | 28653 | 331400.58 | 117 | Ref |
|  | MHO | | MHO | 1415 | 16487.40 | 3 | 0.63(0.20-1.98) |
|  | MHO | | MUO | 1377 | 15944.50 | 8 | 1.43(0.70-2.92) |
|  | MUN | | MUO | 898 | 9922.69 | 7 | 1.55(0.72-3.33) |
|  | MUO | | MHO | 969 | 11071.87 | 11 | 2.79(1.50-5.18) |
|  | MUO | | MUN | 1258 | 14145.55 | 7 | 1.21(0.57-2.60) |
|  | MUO | | MUO | 3763 | 42555.31 | 25 | 1.56(1.01-2.41) |
| Excluding participants who developed UC within the first two years of follow-up | | | | | | | |
| MetS status | Non-MetS | | Non-MetS | 32810 | 378937.71 | 123 | Ref |
|  | Non-MetS | | MetS | 9897 | 112702.52 | 33 | 0.78(0.53-1.14) |
|  | MetS | | Non-MetS | 7021 | 78972.00 | 40 | 1.34(0.93-1.91) |
|  | MetS | | MetS | 12945 | 144264.60 | 74 | 1.32(0.99-1.76) |
| MO status | MHN | | MHN | 29204 | 337234.37 | 107 | Ref |
|  | MHO | | MHO | 1436 | 16723.49 | 3 | 0.69(0.22-2.18) |
|  | MHO | | MUO | 1421 | 16443.23 | 6 | 1.13(0.50-2.58) |
|  | MUN | | MUO | 957 | 10501.51 | 6 | 1.39(0.61-3.16) |
|  | MUO | | MHO | 1003 | 11440.87 | 9 | 2.46(1.24-4.86) |
|  | MUO | | MUN | 1354 | 15153.07 | 9 | 1.60(0.81-3.16) |
|  | MUO | | MUO | 3989 | 44896.29 | 25 | 1.63(1.05-2.52) |
| Excluding participants with less than 1.5 years between baseline (2006-2007) and 2008-2009 surveys | | | | | | | |
| MetS status | Non-MetS | | Non-MetS | 30639 | 352574.67 | 135 | Ref |
|  | Non-MetS | | MetS | 9388 | 106523.63 | 38 | 0.81(0.57-1.17) |
|  | MetS | | Non-MetS | 6594 | 73836.53 | 44 | 1.33(0.95-1.87) |
|  | MetS | | MetS | 12286 | 136297.39 | 89 | 1.44(1.10-1.88) |
| MO status | MHN | | MHN | 27319 | 314344.56 | 117 | Ref |
|  | MHO | | MHO | 1315 | 15249.78 | 4 | 0.87(0.32-2.36) |
|  | MHO | | MUO | 1335 | 15391.64 | 6 | 1.04(0.46-2.37) |
|  | MUN | | MUO | 925 | 10111.93 | 7 | 1.45(0.68-3.11) |
|  | MUO | | MHO | 943 | 10692.16 | 11 | 2.75(1.48-5.10) |
|  | MUO | | MUN | 1271 | 14136.41 | 9 | 1.46(0.74-2.87) |
|  | MUO | | MUO | 3777 | 42390.74 | 27 | 1.61(1.05-2.44) |

Abbreviations: MetS, metabolic syndrome; MO, metabolic obesity; UC, urologic cancer; MHN, metabolically healthy normal weight; MHO, metabolically healthy obesity; MUN, metabolically unhealthy normal weight; MUO, metabolically unhealthy obesity; HR, hazard ratio; CI, conﬁdence interval; Ref, reference.

Model was adjusted for age, gender, smoking status, alcohol consumption, occupation, education level, income, marital status, salt intake and sitting time.
